# Suicidal Ideation Induced by Episodic Cannabis Use

**DOI:** 10.1155/2009/321456

**Published:** 2009-06-11

**Authors:** Michele Raja, Antonella Azzoni

**Affiliations:** ^1^Scuola di Specializzazione in Psichiatria, Università degli Studi di Roma “La Sapienza”, Ospedale “S. Andrea”, 001851 Rome, Italy; ^2^Servizio Psichiatrico di Diagnosi e Cura, Ospedale Santo Spirito, Via Prisciano 26, 00136 Rome, Italy

## Abstract

The report describes a patient who presented suicidal ideation only in two different occasions, immediately after acute cannabis intoxication. He used cannabis only in these two circumstances. Although a definite association between cannabis use and suicidal ideation or behavior has been already reported in the literature, the described case presents two original clinical aspects that deserve consideration. First, episodic assumption of cannabis induced suicidal ideation abruptly. Second, suicidal ideation appeared independent of mood depression, stressors, or life events, suggesting that suicidality may be not a direct consequence of depression and appears to be a relatively independent psychopathological dimension. There seems to be no linear relation between the severity of depression and the risk of suicide.

## 1. Introduction

Usually, cannabis intoxication begins with a “high feeling,” sensation of slowed time, state of relaxation, impaired judgment, and social withdrawal [1]. However, cannabis use can also induce panic, sometimes accompanied by paranoid ideation, more likely in someone who is taking the drug for the first time than in experienced user. Rarely, but especially among new users of cannabis, there occurs an acute depressive reaction [2].

Although, cannabis use has been consistently associated with suicidal ideation and behavior, there is no report on acute suicidality induced by cannabis intoxication. Here, we report a case of suicidal ideation caused by episodic use of cannabis.

## 2. Case Report

A thirty-two years old man entered the emergency room asking for psychiatric consultation. He had smoked cannabis a few hours before. Soon after, he had experienced hopelessness and impulse to commit suicide by fall. Pervasive suicide ideation, anxiety, agitation, and fear were present. In the previous weeks, there had been no mood alterations or psychotic signs. He reported no recent stressful life event and was astonished by his suicidal thinking. He did not see any plausible reason to wish to die. He had never suffered from any significant medical illness or psychiatric disorder. None of his relatives had suffered from psychiatric disorder, substance or alcohol abuse. The patient reported a similar episode of suicidal thought occurred three years before, just after smoking cannabis, spontaneously remitted in a few hours. He claimed to have used cannabis only in these two occasions and was confident in the prompt remission of his current symptoms.

He was admitted to the psychiatric intensive care unit and treated with 4 mg of risperidone and 1000 mg of valproate. Urine toxic screening test revealed the presence of cannabis and no trace of amphetamine, cocaine, opioids, barbiturates, or alcohol. The day after, the patient was free of symptoms and was discharged without therapy.

## 3. Discussion

In patient's life, suicidal ideation presented in two different occasions, only immediately after acute cannabis intoxication. This strongly suggests the causal relationship between intoxication and suicidal ideation.

There is a convincing relationship between suicidal behavior and cannabis use, the latter awakening depressive experiences [3]. Rates of cannabis abuse are elevated among those being treated for depression [4, 5] and among those making a suicidal attempt [6]. In a sample of Italian students, the use of cannabis was associated with suicide risk [7]. In a population of French adolescents, cannabis use appeared to be an independent predictor of suicidal ideation after controlling the depressive symptoms [8]. In a cohort study of young Norwegians, cannabis by itself seemed not to lead to depression but was associated with later suicidal thoughts and attempts [9].

A cross-sectional survey of twin pairs discordant for lifetime cannabis dependence and those discordant for early cannabis use suggests that early cannabis use increases risks for subsequent suicidal attempt [10].

On this background of definite association between cannabis use and suicidal ideation or behavior, the reported case presents two original clinical aspects that deserve consideration. First, episodic assumption of cannabis induced suicidal ideation abruptly. Second, suicidal ideation appeared independent of mood depression, stressors, or life events, suggesting that suicidality may be not a direct consequence of depression and appears to be a relatively independent psychopathological dimension.

There seems to be no linear relation between the severity of depression and the risk of suicide. In some patients, high suicidal risk may be concomitant with relatively minor depressive states, while, in others, extremely severe depression may be immune by suicidal risk [11].
